# Inhalational Alzheimer's disease: an unrecognized—and treatable—epidemic

**DOI:** 10.18632/aging.100896

**Published:** 2016-02-10

**Authors:** Dale E. Bredesen

**Affiliations:** ^1^ Easton Laboratories for Neurodegenerative Disease Research, Department of Neurology, University of California, Los Angeles, CA 90095, USA; ^2^ Buck Institute for Research on Aging, Novato, CA 94945, USA

**Keywords:** mycotoxins, neurodegeneration, cognition, chronic inflammatory response syndrome, biomarkers, dementia, biotoxins

## Abstract

Alzheimer's disease is one of the most significant healthcare problems today, with a dire need for effective treatment. Identifying subtypes of Alzheimer's disease may aid in the development of therapeutics, and recently three different subtypes have been described: type 1 (inflammatory), type 2 (non-inflammatory or atrophic), and type 3 (cortical). Here I report that type 3 Alzheimer's disease is the result of exposure to specific toxins, and is most commonly inhalational (IAD), a phenotypic manifestation of chronic inflammatory response syndrome (CIRS), due to biotoxins such as mycotoxins. The appropriate recognition of IAD as a potentially important pathogenetic condition in patients with cognitive decline offers the opportunity for successful treatment of a large number of patients whose current prognoses, in the absence of accurate diagnosis, are grave.

## INTRODUCTION

Alzheimer's disease is now the third leading cause of death in the United States, following only cardiovascular disease and cancer [1]. There are approximately 5.2 million Americans with AD, but this estimate ignores the many young Americans destined to develop AD during their lifetimes: given the lifetime risk of approximately 15% when including all ApoE genotypes, as many as 45 million of the 318 million Americans now living may develop AD during their lifetimes if no prevention is instituted [2].

Effective treatment of Alzheimer's disease has been lacking, but recently a novel programmatic approach involving metabolic enhancement (MEND) was described, with promising anecdotal results [3]. One of the strategies to optimize treatment development is to identify specific subtypes of Alzheimer's disease that may respond to different optimal programs. Metabolic profiling has revealed three readily distinguishable types of Alzheimer's disease [4]: type 1 is characterized by systemic inflammation, reflected in such laboratory results as a high hs-CRP (high-sensitivity C-reactive protein), low albumin:globulin ratio, and high cytokine levels such as interleukin-1 and interleukin-6. Type 2 is characterized by an atrophic profile, with reduced support from molecules such as estradiol, progesterone, testosterone, insulin, and vitamin D, often accompanied by increased homocysteine and insulin resistance. Type 3 is very dissimilar to the other two types, and may be mediated by a fundamentally different pathophysio-logical process (although, by definition, still β-amyloid positive and phospho-tau positive): the onset is typically younger (late 40s to early 60s); ApoE genotype is usually 3/3 instead of 4/4 or 3/4; the family history is typically negative (or positive only at much greater age); symptom onset usually follows a period of great stress, sleep loss, anesthesia, or menopause/andropause; presentation is not predominantly amnestic but is insteadcortical, with dyscalculia, aphasia, executive dysfunction, or other cortical deficits; and the neurological presentation is often preceded by, or accompanied by, depression. Consonant with these deficits, the imaging studies often indicate extra-hippocampal disease, with more general cerebral atrophy and frontal-temporal-parietal abnormalities on FDG-PET (as opposed to the more restricted, typical temporoparietal reductions in glucose utilization seen in type 1 and type 2). Neuropsychological studies also indicate non-amnestic abnormalities such as executive dysfunction. Laboratory studies often, but not always, feature hypozincemia and/or a high copper:zinc ratio, and may also suggest adrenal fatigue with reduced pregnenolone, DHEA-S (dehydroepiandrosterone sulfate), and/or AM cortisol.

Over the past two decades, elegant work from Dr. R. Shoemaker and his colleagues has demonstrated unequivocally that biotoxins such as mycotoxins are associated with a broad range of symptoms, including cognitive decline (summarized in [5]). These researchers and clinicians identified a constellation of symptoms, signs, genetic predisposition (HLA-DR/DQ haplotypes), and laboratory abnormalities characteristic of patients exposed to, and sensitive to, these biotoxins. The resulting syndrome has been designated chronic inflammatory response syndrome (CIRS). The most common cause of CIRS is exposure to mycotoxins, typically associated with molds such as *Stachybotrys*, *Penicillium*, or *Aspergillus*, present in water-damaged buildings. However, other biotoxins, from the *Borrelia burgdorferi* of Lyme disease or from other tick-borne pathogens, or aquatoxins such as those from dinoflagellates, may also cause CIRS. Laboratory evaluation of CIRS reveals increases in C4a (complement component 4a), TGF- β1 (transforming growth factor beta-1), MMP9 (matrix metalloprotease 9), specific cytokines, and decreases in MSH (melanocyte-stimulating hormone), VEGF (vascular endothelial growth factor), and ADH (anti-diuretic hormone), as well as frequent hypercortisolemia, hypozincemia, and other abnormalities. These laboratory abnormalities are frequently accompanied by nasal colonization by MARCoNS (multiple-antibiotic-resistant coagulase-negative *Staphylococcus*) and compromised visual contrast sensitivity. Specific HLA-DR/DQ haplotypes are associated with sensitivity to mycotoxins, and thus account for the vast majority of CIRS cases.

Most importantly, Dr. Shoemaker and his colleagues developed an effective therapeutic regimen for CIRS. This is a complicated, multi-step regimen that includes toxin binding by cholestyramine, treatment of MARCoNS by BEG (Bactroban, EDTA, and gentamicin) nasal spray, intranasal VIP (vasoactive intestinal peptide), removal of toxin source, addressing all of the hormonal, gastrointestinal, and other biochemical abnormalities (e.g., anti-diuretic hormone administration if indicated), and follow-up laboratory tests to ensure return to normal.

Here I report that type 3 Alzheimer's disease is a phenotypic manifestation of CIRS. Both may present with cognitive decline that goes beyond a restricted amnestic presentation to include executive dysfunction and other deficits; as well as depression, hypozincemia, hypersensitivity to stress, and dysfunction of the hypothalamic-pituitary-adrenal (HPA) axis. In the description of the three types of Alzheimer's disease [4], it was pointed out that all of the initial six patients described with type 3 Alzheimer's disease had a significant history of toxic exposures, including in some cases mycotoxins. Follow-up evaluation of these and other patients with type 3 Alzheimer's disease revealed that a majority of these patients meet criteria for CIRS.

## RESULTS

### Case studies

#### Patient 1

This is a follow-up description of the first patient described in a previous report [4]. A 52-year-old woman presented with a two-year history of cognitive decline, beginning with dyscalculia. Her cognitive decline had been preceded by severe stress with employment changes, menopause at 51 years of age, and four episodes of general anesthesia for relatively minor procedures. She declined over several months and developed a simple, childlike affect. Despite these symptoms, she learned and remembered the names of all 28 children on the playground at her son's school. Family history was negative for dementia. Her MoCA score was 19/30. Her MRI showed global cerebral and cerebellar atrophy. There were several areas of FLAIR (fluid-attenuated inversion recovery) hyperintensity in the subcortical and periventricular white matter. An amyloid PET (positron emission tomography) scan was positive. Her CSF (cerebrospinal fluid) included reduced Aβ42 of 294pg/ml and increased p-tau of 133pg/ml, strongly supporting the diagnosis of Alzheimer's disease.

BMI was 24.9. ApoE was 3/3, klotho variant negative (SNP Rs9536314), hs-CRP 1.4mg/l, albumin:globulin ratio 1.57, IL-6 1.4pg/dl, hemoglobin A1c 5.3%, fasting insulin 4.5mIU/l, TSH 2.14mIU/l, free T3 4.2pg/ml, reverse T3 11ng/dl, free T4 1.0pg/ml, progesterone < 0.21ng/ml, estradiol 3pg/ml, 17-hydroxypregnenolone 14ng/dl, AM cortisol 9mcg/dl, 25-hydroxycholecalciferol 22ng/ml, total cholesterol 264mg/dl, HDL-cholesterol 67mg/dl, LDL-cholesterol 167mg/dl, triglycerides 61mg/dl, cholesterol:HDL ratio 3.7, serum copper 101mcg/dl, serum zinc 56mcg/dl, and Cu:Zn ratio 1.8. Heavy metal evaluation from blood was negative.

She was started on the MEND protocol [3], and because of the presentation consistent with type 3 AD, further history was obtained and additional laboratory evaluation undertaken. She had moved into a new home two years before the onset of symptoms. Her symptoms tended to worsen when she returned home from travel. Evaluation of the home by a mold expert isolated *Stachybotrys*, *Penicillium*, and *Aspergillus*, all mold genera associated with neurotoxin production. Her C4a was 22,799ng/ml (normal <2800ng/ml), TGF- β1 (transforming growth factor beta-1) was 6660pg/ml (normal <2382pg/ml), and MMP9 (matrix metalloprotease 9) was 620ng/ml (normal <332ng/ml). Serum test for Lyme disease was negative. Her nasal cavity was positive for MARCoNS (multiple-antibiotic-resistant coagulase-negative *Staphylococcus*), a common finding associated with CIRS. She underwent visual contrast sensitivity testing, which she failed. Since these findings, taken together, are strongly suggestive of a diagnosis of CIRS, she was started on the Shoemaker Protocol [5]. After five weeks on this protocol, she began to show modest subjective improvement, and her C4a had decreased to 2750ng/ml.

Comment: This patient fulfilled diagnostic criteria for both Alzheimer's disease and CIRS. She presented in a manner typical for type 3 Alzheimer's disease, with a non-amnestic onset (although her memory was affected later in the course), multiple stresses, onset at a young age (50 years of age), in association with hormonal reduction, ApoE ε4-negative genotype, hypozincemia, hypothalamic-pituitary-adrenal axis (HPA) abnormality, and negative family history. Her laboratory values were strongly supportive of CIRS, with a very high C4a, as well as high TGF-β1 and MMP9, and the presence of MARCoNS, as well as reduced visual contrast sensitivity (VCS). Typical for the patients in this report (and those with CIRS in general), the chronic inflammation was not accompanied by a marked elevation of hs-CRP, although the value of 1.4mg/l represents a slight elevation above normal. The inciting agents for the CIRS in her case are likely to have been mycotoxins, based on the presence of *Stachybotrys*, *Penicillium*, and *Aspergillus* in the patient's home and the negative test for Lyme disease. These mycotoxins may include trichothecenes (from *Stachybotrys*), aflatoxin (from *Aspergillus*), and ochratoxin A (from *Aspergillus* and *Penicillium*), among others, all of which exhibit well-described neurotoxic effects. In addition, inflammagens, microbes, microbial fragments, endotoxins, lipopolysaccharides, and biologically-produced VOCs (volatile organic compounds) may have contributed to the CIRS [6]. It is noteworthy that, as has been described repeatedly with CIRS, the mold exposure had not been suspected by patient, family, or healthcare providers prior to the recognition of the syndrome. At the current time, it is too early to tell how much improvement the patient will experience with the Shoemaker Protocol, but she has begun to show modest improvement, which is in stark contrast to her severe decline over the previous two years.

#### Patient 2

This is a follow-up description of the second patient described in a previous report [4]. A 59-year-old man began to note word-finding difficulties, followed by difficulties with arithmetic. These symptoms had been preceded by depression for seven years. He had been a type A personality with a high-powered position, whose neurological symptoms had begun after two years of the most stressful time of his career. His personality changed, and he became passive and timid. Neuropsychological testing showed profound impairment in semantic fluency, executive functioning, attention, overall mental status, processing, and visual memory. Fluorodeoxyglucose PET scan showed reduced metabolism in temporal and parietal lobes, left > right precuneus, and left frontal lobe. A diagnosis of Alzheimer's disease was made at a nationally recognized dementia clinic.

His BMI was 24.9, ApoE genotype 2/3, hs-CRP 0.5mg/l, albumin 4.5g/dl, globulin 2.4g/dl, albumin:globulin ratio 1.9, AM cortisol 15.8mcg/dl, total cholesterol 235mg/dl, HDL-cholesterol 70mg/dl, LDL-cholesterol 150mg/dl, triglycerides 75mg/dl, cholesterol:HDL ratio 3.4, DHEA-S 130mcg/dl, progesterone 0.4ng/ml, fasting insulin 6mIU/l, 25-hydroxycholecalciferol 44.5ng/ml, alpha-tocopherol 22.5mg/l, beta-gamma-tocopherol 0.5mg/l, TSH 2.98mIU/l, free T3 2.7ng/ml, free T4 1.2ng/dl, reverse T3 21ng/dl, pregnenolone <5ng/dl, homocysteine 7.3μmol/l, folate 16.6ng/ml, RBC Mg 5.5mg/dl, serum iron 135mcg/dl, serum copper 97mcg/dl, serum zinc 59mcg/dl, Cu:Zn ratio 1.6, blood arsenic/lead/mercury all <2mcg/l, TNF 1.2pg/ml, and IL-6 1.7pg/ml.

A sleep study showed mild obstructive sleep apnea, with an apnea/hypopnea index of 7 events per hour. No REM behavioral disturbance was noted.

Because of the presentation consistent with type 3 Alzheimer's disease, further history was obtained and additional laboratory evaluation was undertaken. He had spent time in foreclosed homes that had suffered water damage. His HLA-DR/DQ (Table 1) was 12-3-52B, which is uncommon (less than 5% of the population) and strongly associated with hyper-sensitivity to biotoxins [5]. His TGF-β1 was elevated at 9040pg/ml, and C4a was elevated, as well. His nasal cavity was colonized by MARCoNS, an association of CIRS. Because his history and laboratory evaluation were suggestive of a diagnosis of CIRS, he was treated with the Shoemaker Protocol [5], and began to show subjective improvement in cognition after several weeks.

**Table 1:** HLA-DR/DQ haplotypes in patients with type 3 Alzheimer's disease

Age at symptom onset (years)	Major symptoms	HLA-DR/DQ	Comment
50	Dyscalculia, executive	10-3-52 B10-5 (low MSH)	ApoE3/3
54	Executive, visual	11-3-52B ** 7-2-53 *	ApoE3/3
72	Executive, dyscalculia	4-3-53 ** 15-6-51 * (Lyme)	ApoE3/3
65	Spatial > verbal memory, attention, irritability, depression	11-3-52B ** 13-6-52B *	ApoE3/3
54	Executive, visuospatial, memory, depression	17-2-52A * 1-5 (low MSH)	ApoE4/4
59	Aphasia, executive, dyscalculia, depression	12-3-52B ** 15-6-51 * (Lyme)	ApoE2/3
59	Headache, executive	4-3-53 ** 15-6-51 * (Lyme)	ApoE ND
66	Headache, executive, memory	11-3-52B ** 13-6-52C *	ApoE ND

* Pathogen-specific HLA-DR/DQ-related sensitivity (mold or Lyme).

** Multiple-biotoxin-sensitive HLA-DR/DQ association.

Comment: This patient was typical both in his presentation of type 3 Alzheimer's disease and in his presentation of CIRS. His relatively rapid initial response to treatment with the Shoemaker Protocol supports the diagnosis of CIRS and the relationship between type 3 Alzheimer's disease and CIRS.

#### Patient 3

A 72-year-old man who had been a high school mathematics state champion with an IQ of 164 began to have word-finding difficulty, as well as dyscalculia. There was no family history of dementia except in his mother, who in her 90s had undergone cognitive decline. Genetic analysis disclosed his ApoE genotype as 3/3. His neuropsychological testing was suggestive of subcortical dementia, and thus Alzheimer's disease was initially felt to be unlikely.

However, his CSF showed reduced Aβ42 and increased p-tau, strongly suggestive of Alzheimer's disease.

His homocysteine was 10.4μM, hs-CRP 0.2mg/l, albumin 4.6g/dl, albumin:globulin ratio 2.0, 25-hydroxycholecalciferol 32.5ng/ml, alpha-tocopherol 13.4mg/l, hemoglobin A1c 5.4%, fasting insulin 5.6mIU/l, total cholesterol 167mg/dl, HDL cholesterol 94mg/dl, LDL cholesterol 62mg/dl, triglycerides 56mg/dl, TSH 1.0mIU/l, free T4 1.29ng/dl, reverse T3 23.2ng/dl, AM cortisol 21.7ug/dl, pregnenolone 48ng/dl, and RBC magnesium 4.9mg/dl.

Because of his presentation suggestive of type 3 AD (his advanced age compared to other patients notwithstanding), he underwent HLA-DR/DQ testing, which revealed that he carried the uncommon, multiple biotoxin sensitivity haplotype 4-3-53. Urine testing was positive for mycotoxins.

Comment: This patient was atypical for type 3 Alzheimer's disease in his late age of onset, but was in all other respects typical, including a cortical presentation with aphasia and dyscalculia, ApoE ε4-negative genotype, lack of family history (except in the tenth decade), neuropsychological testing suggesting an atypical presentation for Alzheimer's, with CSF indicative of Alzheimer's disease. His high reverse T3 and high AM cortisol are both suggestive of ongoing stress, and his HLA-DR/DQ is supportive of CIRS given the uncommon, multiple biotoxin-sensitive haplotype and the positive urine test for mycotoxins.

#### Patient 4

A 54-year-old man developed depression after 70% of his company's employees were laid off. He was treated with an antidepressant, and three years later began to have difficulty understanding the difference between left-turn lanes, failing to appreciate the difference between the more acute left turn (far left lane) and the more gentle left turn (second lane from the left), leading to lane crosses and near accidents. He then developed executive, visuospatial, and memory deficits. He was unable to organize, unable to understand how to set the table, had difficulty with drawing and writing, and was unable to work. He was unable to pay bills, and had difficulty speaking multi-syllabic words.

He was evaluated at a university dementia center, where he was found to carry the ApoE4/4 genotype, his MMSE (mini-mental status exam) was 24, and a diagnosis of early onset Alzheimer's disease was made. He was treated with donepezil, to which he responded with improvement, and memantine was later added. He was also involved in a clinical trial in which he was given an antibody to amyloid-beta peptide, and his wife noted that each time he received the antibody, he declined markedly, with several days of severe confusion and non-communication, followed by a slow return to baseline. Over the ensuing two years he declined rapidly, and his MoCA (Montreal Cognitive Assessment) score at that time was 6/30.

His wife and he had lived in the same home for 19 years. She had suffered from asthma during part of that time. Stachybotrys, Penicillium, and Aspergillus were identified in the home by ERMI (environmental relative moldiness index) testing. His TGF-β1 was elevated at 3260pg/ml, and his HLA-DR/DQ was 17-2-52A, a mold-sensitive haplotype. His Borrelia burgdorferi Western blot was negative. His methylmercury was markedly elevated, twice the 95th percentile, and inorganic mercury was at the 90th percentile. His BMI was 22, fasting glucose 90mg/dl, hemogloblin A1c 5.5%, fasting insulin 2.2 mIU/l, homocysteine 15.1μM, vitamin B12 333pg/ml, folate 9.8ng/ml, 25-hydroxycholecalciferol 45.9ng/ml, zinc 78mcg/dl, copper 73mcg/dl, free T3 2.1pg/ml, free T4 1.23ng/dl, reverse T3 19.7ng/dl, TSH 4.0uIU/ml, AM cortisol 17.7mcg/dl, total cholesterol 231mg/dl, LDL cholesterol 110mg/dl, HDL cholesterol 112mg/dl, and triglycerides 43mg/dl. Cyrex Arrays 2 (for gastro-intestinal permeability), 3 (for gluten sensitivity), and 20 (for blood-brain barrier permeability) were negative.

Comment: This patient presented with typical type 3 Alzheimer's disease, with the single exception that his ApoE genotype was not ε4-negative. In searching for potential toxic etiologies, both mycotoxins and mercury were identified as candidates. His mold-sensitive haplotype, 17-2-52A, along with elevated TGF-β1 and cognitive decline, coupled with definitive evidence of molds within the home, all support a diagnosis of CIRS.

#### Patient 5

A 50-year-old woman experienced depression following a hysterectomy, despite hormone-replacement therapy (although resulting hormone levels were not determined, so it is unknown whether optimal levels were achieved). Four years later she began to have word-finding difficulty, disorientation, difficulty driving, difficulty following recipes and other instructions, and increased depression following her son's leaving home. Her husband noted that she improved markedly following several days of rest, and declined markedly with sleep deprivation, viral illness, or stress. On neuropsychological evaluation, it was noted that she could not remember her own family history (which, based on the recall of knowledgeable others, was negative for dementia), that she exhibited paucity of speech, poor semantic fluency, confabulation on memory tests, and that she was anosmic. The diagnostic impression was of frontal, temporal, and parietal deficits. Her MRI was read as normal, but quantitative volumetrics were not performed. Her FDG-PET was abnormal, with reduced glucose utilization in the parietotemporal regions, and to a much lesser extent in the frontal region.

Her BMI was 18, ApoE genotype was 3/3, hs-CRP 0.2mg/l, homocysteine 8μM, fasting insulin 4.2uIU/ml, hemoglobin A1c 5.1%, free T3 2.1pg/ml, free T4 1.33ng/dl, reverse T3 23ng/dl, fT3:rT3 9, TSH 1.16uIU/ml, progesterone 0.3ng/ml, AM cortisol 7.2mcg/dl, pregnenolone 19ng/dl, 25-hydroxychole-calciferol 37ng/ml, vitamin B12 799pg/ml, alpha-tocopherol 12.5mg/l, zinc 82mcg/l, copper 99mcg/l, copper:zinc ratio 1.2, ceruloplasmin 20mg/dl, total cholesterol 221mg/dl, HDL cholesterol 67mg/dl, non-HDL cholesterol 167mg/dl, triglycerides 82mg/dl, urinary mercury:creatinine < 2.8, Lyme antibodies negative, C4a 5547ng/ml, and TGF-β1 7037pg/ml.

She was treated with donepezil, to which she responded well, and duloxetine, which reduced her depression. However, her cognitive function continued to decline.

Comment: This patient's presentation was typical for type 3 Alzheimer's disease in age of onset, preceding depression, ApoE non- ε4 genotype, executive dysfunction, predominantly non-amnestic onset, negative family history, and strong response to stress and sleep. Her failure to recall her own family history, demonstrating the loss of long-term storage or recall, rather than a restriction to recent memory, is also typical. Patients with type 3 Alzheimer's disease often have relatively low triglyceride levels (in the 40-70 range), and this patient's higher triglyceride level of 82 may have been associated with her hypothyroidism. Although the copper:zinc ratio is not as high as for most patients with type 3 Alzheimer's disease, the free copper (estimated by serum copper minus three times ceruloplasmin) is high. The high reverse T3 and low fT3:rT3 ratio of 9 (normal > 20) both suggest ongoing stress, and the low AM cortisol and low pregnenolone are compatible with HPA axis dysfunction. The low progesterone shows that her hormone-replacement therapy is suboptimal, a potential contributor to both type 3 Alzheimer's disease and CIRS. CIRS is suspected based on the high C4a; the negative Lyme antibody titer, along with lack of exposure to dinoflagellates and other water-borne CIRS-related agents, suggests that the most likely cause of CIRS in this patient is mycotoxins.

#### Patient 6

A 54-year-old woman began to have difficulty driving at night, followed by difficulty writing numbers, along with exhaustion. This was initially ascribed to menopause. She was unable to complete her work in a timely fashion, and had to check her work many times over because of a propensity to make mistakes, all of which was highly unusual for her. She had difficulty with organization and with visual recognition, including difficulty reading. She had to resign from work, and this led to severe stress.

There was no family history of dementia, and her ApoE genotype was 3/3. Her MoCA was 23, with 4/5 on memory (5/5 on a subsequent test) but missing all serial 7s except the first (which she missed on a subsequent test), and missing the clock numbers and hands, cube copying, as well as part of one of the repeated sentences. Her primary care provider made the diagnosis of MCI. A brain MRI showed mild atrophy, without hippocampal predilection, and a second MRI, two and one-half years later, showed slightly more severe generalized atrophy. A neurologist noted mild EEG abnormalities and therefore prescribed anticonvulsants, which had no noticeable effect on her condition.

Because of the presentation typical for type 3 Alzheimer's disease, further evaluation was undertaken, revealing that her HLA-DR/DQ haplotypes were 11-3-52B (uncommon, multiple-biotoxin sensitive) and 7-2-53 (mold sensitive). Her TGF-β1 was elevated at 5780pg/ml (normal 344-2382pg/ml). She failed a visual contrast sensitivity (VCS) test.

Comment: This patient's presentation was typical for type 3 Alzheimer's disease, and the combination of elevated TGF-β1 and HLA-DR/DQ multiple-biotoxin sensitive and mold-sensitive haplotypes, along with a failed VCS test, supports the diagnosis of CIRS.

#### Patient 7

A 64-year-old man began to complain of headache, leg cramps, irritability, distractibility, and difficulty with memory. Evaluation noted in addition a peripheral neuropathy and hyposmia. Neuro-psychological assessment revealed a high-functioning individual with mild reductions in spatial > verbal memory. CT angiogram did not disclose a source for the headaches, and an MRI revealed generalized cerebral atrophy and areas of FLAIR (fluid-attenuated inversion recovery) hyperintensity. A diagnosis of amnestic mild cognitive impairment was made, and over the next seven years his headaches abated but cognitive decline progressed slowly, and he became frustrated, irritable, and occasionally depressed.

His fasting glucose was 101mg/dl, hemoglobin A1c 5.4%, fasting insulin 3mIU/l, homocysteine 10.6μM, vitamin B12 543pg/ml, hs-CRP 1.1mg/l, albumin:globulin ratio 1.8, free T3 2.8pg/ml, free T4 1.2pg/ml, TSH 1.19mIU/l, vitamin D 37ng/ml, total cholesterol 191mg/dl, HDL 92mg/dl, LDL 91mg/dl, triglycerides 45mg/dl, serum copper 97mcg/dl, serum zinc 57mcg/dl, copper:zinc ratio 1.7. His HLA-DR/DQ haplotypes were 11-3-52B, a multiple-biotoxin-sensitive haplotype, and 13-6-52B, a mold-sensitive haplotype. His TGF-β1 was markedly elevated at 20,657, and MMP9 was 684. Nasopharyngeal culture was positive for MARCoNS. Evaluation of his home revealed *Penicillium* and *Aspergillus*. His Cyrex Arrays 2, 3, 5, and 20 were all abnormal: Cyrex Array 2 revealed IgM anti-occludin/zonulin of 2.5 (0.1-2.1), indicative of gastrointestinal hyperpermeability; Cyrex Array 3 revealed a high level of IgA anti-omega gliadin; Cyrex Array 5 disclosed a high level of auto-antibodies to myelin basic protein and glutamic acid decarboxylase 65; and Cyrex Array 20 was compatible with hyper-permeability of the blood-brain barrier.

Comment: This patient was atypical for type 3 AD in that he presented with single domain, amnestic MCI. However, his other symptoms and laboratory values supported both a diagnosis of CIRS and a diagnosis of type 3 AD: his headache, depression, irritability, markedly elevated TGF-β1, HLA haplotypes characteristic of biotoxin sensitivity (11-3-52B) and mold sensitivity (13-6-52B), positive nasopharyngeal culture for MARCoNS, autoantibodies, and the presence of *Aspergillus* and *Penicillium* in his home, are all compatible with a diagnosis of CIRS. His depression, difficulty focusing and keeping his train of thought, hypozincemia, hypotriglyceridemia, and general atrophy on MRI with areas of FLAIR hyperintensity, are all compatible with type 3 AD.

## DISCUSSION

These findings suggest that patients with presentations compatible with type 3 Alzheimer's disease should be evaluated for CIRS (as well as other toxic exposures, such as mercury and copper). These are treatable etiologic agents, and thus treatable causes of Alzheimer's disease. Furthermore, it may be particularly important to identify or exclude these toxins in patients with type 3 Alzheimer's disease since amyloid may be protective against toxins, especially metals, so reducing the amyloid burden without reducing the toxic exposure may potentially exacerbate the pathophysiology (and, indeed, may have done so in patient 4). Conversely, the exclusion of patients with type 3 Alzheimer's disease may potentially enhance the group efficacy of anti-amyloid therapies. While the patients described here are still too early in their courses to know the final therapeutic outcome, it is important to note that improvement in cognitive decline has been observed routinely in the treatment of CIRS with the Shoemaker Protocol.

It is noteworthy that a recent report described the direct detection of fungi in the brains of patients who had died with Alzheimer's disease, contrasting with a lack of detection of fungi in control brains [7]. This finding raises the possibility that the mycotoxic effects that occur in CIRS associated with type 3 Alzheimer's disease may be accompanied by active infection. However, unlike in the case of CIRS, there is as yet no indication that treating the putative fungal infection has any ameliorative effect on the cognitive decline (unless a specific diagnosis such as Cryptococcal meningitis is made).

The increasing number of reports of various pathogens identified in the brains of patients with Alzheimer's disease—from viruses such as *Herpes simplex* [8] to oral bacteria such as *P. gingivalis* [9] to fungi such as *C. glabratus* [7]—raises the possibility that what is referred to as Alzheimer's disease may actually be the result of a protective response to various brain perturbations. Indeed, the three different types of Alzheimer's disease that are distinguishable based on metabolic profiling fit well with the three known effects of amyloids such as Aβ: Aβ is produced in response to infection, and exhibits antimicrobial effects [10]; furthermore, Aβ is a component of the inflammatory response, with NFκB inducing proteases involved in its formation. These inflammatory/antimicrobial effects are prominent in type 1 AD, in which systemic inflammation is reflected in high hs-CRP levels, reduced albumin:globulin ratios, and increased cytokine levels. In contrast, Aβ is also produced in response to the withdrawal of trophic support [11], and indeed APP processing to Aβ peptides is influenced by factors involved in trophic support, such as estradiol and SirT1. This atrophic response is compatible with the metabolic profile in type 2 Alzheimer's disease, in which hormonal support is typically reduced and the biochemical mediators of systemic inflammation are not increased.

Thus amyloid may function as part of an inflammatory/antimicrobial response or as part of an atrophic response; however, the third major cause of amyloid production is as part of an anti-toxin response, especially as part of the response to the toxic accumulation of divalent metals such as mercury, copper, or iron [12]. The liberation of amyloid as part of a response to toxins forms the underpinning of type 3 Alzheimer's disease. Although some of these patients did indeed demonstrate laboratory values compatible with metal toxicity, the majority, including the seven described above, had instead, histories, exposures, genetics, laboratory values, visual contrast sensitivities, and at least initial, subjective therapeutic responses that were all compatible with a diagnosis of CIRS.

Interestingly, the pathophysiology of CIRS includes effects that are relevant to all three types of Alzheimer's disease: chronic inflammation is produced by the ongoing activation of the innate immune system, infection is at least theoretically possible due to the exposure to mold species and other aerosol-derived microbes, trophic support may be reduced due to pathogen-derived proteases, and, as noted above, multiple different mycotoxins may be included in the aerosols of water-damaged buildings. Therefore, it is perhaps not surprising that CIRS may be causally associated with Alzheimer's disease.

Since the majority of patients with CIRS have a combination of genetic sensitivity plus exposure to a complex aerosolic mixture of mycotoxins, spores, bacteria, microbial fragments, inflammagens, volatile organic compounds, and other molecular species, the majority of patients with type 3 Alzheimer's disease and CIRS are likely to have an inhalational cause of Alzheimer's disease (IAD). This recognition may be critical to optimizing therapeutic approaches to patients with this form of Alzheimer's disease.

It is noteworthy that patients with IAD did not in most cases exhibit extraneural symptoms of CIRS, such as asthma, pruritus, rhinorrhea, chronic fatigue, epistaxis, dyspnea on exertion, hemoptysis, diarrhea, vomiting, loss of appetite, alopecia, chronic sinus infections, chronic bronchitis, arthralgias, or otitis. This raises the obvious question of how patients who exhibit the laboratory abnormalities, genetics, and exposures characteristic of CIRS might develop neural abnormalities yet, in large measure, escape the characteristic extraneural manifestations. There are several possible explanations: one possibility is that the autoimmune component of the response may be more significant in the typical CIRS patients than in the IAD patients, or that the overall innate system immune stimulation (ISIS) characteristic of CIRS may be more active in the typical CIRS patients. Another possibility is that the genetics may favor a chronic neurodegenerative syndrome over a more typical immune-mediated systemic illness. Evaluation of the HLA-DR/DQ haplotypes in eight patients with IAD may provide some support for this possibility: whereas about 95% of CIRS patients display one of the four multiple-biotoxin-sensitive haplotypes (4-3-53, 11-3-52B, 12-3-52B, 14-5-52B) or one of the seven mold-sensitive haplotypes (7-2-53, 7-3-53, 13-6-52A, 13-6-52B, 13-6-52C, 17-2-52A, 18-4-52A), six of the eight IAD patients displayed both a multiple-biotoxin-sensitive haplotype and a pathogen-sensitive (mold or Lyme) haplotype (of the other two, one was an ApoE4 homozygote and the other was an ApoE4 heterozygote with a mold-sensitive haplotype). Based on the frequencies of these haplotypes, the chance of picking eight people at random and having six of them display both one of the uncommon multiple-biotoxin-sensitive haplotypes and one of the pathogen-sensitive haplotypes is less than one in one million. Finally, a third possibility is that the neurodegenerative phenotype may represent a late-stage effect, analogous to the tertiary lues syndromes, whereas the typical CIRS symptoms may be analogous to the primary and secondary syndromes. These three possibilities are not mutually exclusive.

As noted previously, type 3 Alzheimer's disease is readily distinguished from types 1 and 2 biochemically, genetically, and symptomatically [4]. Given previous descriptions of cortical presentations in Alzheimer's disease, as well as the current studies, it is possible that type 3 represents on the order of 10% of patients with Alzheimer's disease, thus potentially affecting hundreds of thousands of Americans. This percentage would be much higher for the subgroup of patients who are ApoE4-negative and whose symptoms begin prior to the age of 65. This potential epidemic may have gone unrecognized to date for several reasons: (1) because it has been hidden beneath the large umbrella of Alzheimer's disease diagnoses grouped without respect to metabolic profiling-based type; (2) because CIRS is neither widely recognized nor typically considered in evaluations at neurological centers specializing in dementia; and (3) because standard evaluations for patients presenting with dementia or mild cognitive impairment do not include laboratory testing for the innate system immune stimulation (ISIS) that is characteristic of CIRS.

There are many cases of CIRS without dementia, and conversely, there are examples of type 3 Alzheimer's disease without CIRS. Nonetheless, the concordance of symptoms and laboratory values suggests an important and potentially extensive overlap. Given the relatively common presentation of AD with cortical symptoms, it is possible that CIRS may contribute to a significant minority of patients with AD. It is unlikely that the coexistence of type 3 Alzheimer's disease and CIRS described here is simply a coincidence: although both Alzheimer's disease and CIRS are relatively common illnesses, the finding that most patients with type 3 Alzheimer's disease also have laboratory abnormalities typical of CIRS, the repeated finding of biotoxin-sensitive HLA-DR/DQ haplotypes in type 3 Alzheimer's disease patients, the discovery of well-described neurotoxin-producing mold genera in the homes of these individuals, the initial response to treatment, and the similarity of the symptoms all argue that type 3 Alzheimer's disease is most commonly IAD, a phenotypic manifestation of CIRS.

It is not yet clear why IAD occurs in a subset of CIRS patients but not in all. It is possible that genotype plays a role, or that specific mycotoxins or inflammagens or other inciting agents may predispose to this phenotypic manifestation of CIRS, or that other contributing factors, such as metal toxicity or stress or HPA axis dysfunction, may increase risk for IAD with CIRS. It is also possible that a combination of these factors may be critical in the development of this syndrome.

Since the toxins associated with IAD are addressable therapeutically, it will be important to recognize and evaluate candidates appropriately. IAD should be suspected if more than two of the characteristics listed in Table 2 are present in a patient with Alzheimer's disease, mild cognitive impairment, or subjective cognitive impairment.

**Table 2:** Symptoms, signs, and laboratory values suggestive of type 3 Alzheimer's disease

Characteristic	Comment
Age at symptom onset less than 65 years.	Symptoms often begin in the 50s or late 40s.
ApoE ε4-negative genotype.	Typically ApoE3/3 unless there are other risk factors.
Negative family history or family history positive with symptom onset only in much older individuals than the patient.	
Symptom onset in association with menopause or andropause.	
Depression as a preceding or significant accompaniment of the cognitive decline.	
Headache as an early or preceding symptom.	
Atypical presentation, in which memory consolidation is not the initial and dominant characteristic.	Typical deficits include executive deficits, dyscalculia, paraphasias, or aphasia.
Precipitation or exacerbation by a period of great stress (e.g., loss of employment or marriage dissolution or family change) and sleep loss.	The degree of dysfunction is also markedly affected by stress and sleep loss.
Exposure to mycotoxins or metals (e.g., inorganic mercury via amalgams, or organic mercury via the consumption of large fish such as tuna) or both.	
Diagnosis of CIRS with cognitive decline.	Cognitive decline is common with CIRS.
Imaging suggestive of more than typical Alzheimer's involvement.	FDG-PET may show frontal as well as temporoparietal reductions in glucose utilization, even early in the course of the illness; MRI may show generalized cerebral and cerebellar atrophy, especially with mild FLAIR (fluid-attenuated inversion recovery) hyperintensity.
Low serum triglycerides or triglyceride:total cholesterol ratio.	Triglycerides are often in the 50s.
Low serum zinc (<75mcg/dl) or RBC zinc, or high copper:zinc ratio (>1.3).	
HPA axis dysfunction, with low pregnenolone, DHEA-S, and/or AM cortisol.	
High serum C4a, TGF-β1, or MMP9; or low serum MSH (melanocyte-stimulating hormone). Positive deep naso-pharyngeal culture for MARCoNS.	See reference 5.
HLA-DR/DQ associated with multiple biotoxin sensitivities or pathogen-specific sensitivity.	See reference 5.

The appropriate recognition of IAD as a potentially important pathogenetic condition in patients with cognitive decline offers the opportunity for successful treatment of a large number of patients whose current prognoses, in the absence of accurate diagnosis, are grave. Furthermore, the possibility that inhalational factors may contribute not only to the clinically distinct syndrome of IAD but also, partially, to typical type 1 or 2 Alzheimer's disease, or to other neurodegenerative diseases, should be considered in the evaluation of these patients.
